# Duration of frequent or severe respiratory tract infection in adults before diagnosis of IgG subclass deficiency

**DOI:** 10.1371/journal.pone.0216940

**Published:** 2019-05-21

**Authors:** James Barton, Clayborn Barton, Luigi Bertoli

**Affiliations:** 1 Southern Iron Disorders Center, Birmingham, Alabama, United States of America; 2 Department of Medicine, University of Alabama at Birmingham, Birmingham, Alabama, United States of America; 3 Department of Medicine, Brookwood Medical Center, Birmingham, Alabama, United States of America; Central University of Tamil Nadu, INDIA

## Abstract

Many adults with IgG subclass deficiency (IgGSD) experience long intervals of frequent/severe respiratory tract infection before IgGSD diagnosis, but reasons for delays in IgGSD diagnoses are incompletely understood. We performed a retrospective study of 300 white adults (ages ≥18 y) with IgGSD including frequency analyses of age at IgGSD diagnosis, duration of frequent/severe respiratory tract infection before IgGSD diagnosis, and age at onset of frequent/severe infection (calculated). We performed multivariable regressions on age at diagnosis, infection duration, and age at infection onset using these variables, as appropriate: sex; age at diagnosis; diabetes; autoimmune condition(s); atopy; allergy; corticosteroid use; body mass index; serum immunoglobulin isotype levels; blood lymphocyte subsets; three IgGSD-associated human leukocyte antigen-A and -B haplotypes; and referring physician specialties. Mean age at diagnosis was 50 ± 12 (standard deviation) y (median 50 y (range 19–79)). There were 247 women (82.3%). Mean infection duration at IgGSD diagnosis was 12 ± 13 y (median 7 y (range 1–66)). Mean age at infection onset was 38 ± 16 y (median 38 y (range 4, 76)). Age at infection onset was ≥18 y in 95.7% of subjects. Regressions on age at diagnosis and infection duration revealed no significant associations. Regression on age at infection onset revealed one positive association: age at diagnosis (p <0.0001). We conclude that the median duration of frequent/severe respiratory tract infection in adults before IgGSD diagnosis was 7 y. Older adults may be diagnosed to have IgGSD after longer intervals of infection than younger adults. Duration of frequent/severe respiratory tract infection before IgGSD diagnosis was not significantly associated with routine clinical and laboratory variables, including referring physician specialties.

## Introduction

Immunoglobulin (Ig) G subclass deficiency (IgGSD) in adults is characterized by frequent or severe respiratory tract infection, suboptimal IgG response to polyvalent pneumococcal polysaccharide vaccination, female predominance, and increased prevalence of autoimmune conditions [1–3]. Many adults with IgGSD experience long intervals of frequent or severe respiratory tract infection before IgGSD diagnosis [4–6]. Delays in diagnosis of IgGSD in adults have been associated with greater morbidity due to major infections [6–8]. We postulated that analyses of age at IgGSD diagnosis, duration of frequent or severe respiratory tract infection before IgGSD diagnosis, and age at onset of frequent or severe respiratory tract infection could provide insights into the chronology of IgGSD manifestations and delays of IgGSD diagnosis in adults.

To learn more, we performed a retrospective study of 300 unrelated non-Hispanic whites diagnosed to have IgGSD as adults (ages ≥18 y). We analyzed distributions of ages at IgGSD diagnosis, duration of frequent or severe respiratory tract infection before IgGSD diagnosis, and ages at onset of frequent or severe respiratory tract infection. We also performed multivariable regressions on age at IgGSD diagnosis, duration of frequent or severe respiratory tract infection, and age at onset of frequent or severe respiratory tract infection using these independent routine clinical and laboratory variables, as appropriate: sex; age at diagnosis; diabetes; autoimmune condition(s); atopy; allergy; corticosteroid use; body mass index; serum Ig isotype levels; blood lymphocyte subsets; three human leukocyte antigen (HLA)-A and -B haplotypes associated with IgGSD in adults; and specialties of referring physicians. Herein, we determined that the median duration of frequent/severe respiratory tract in adults before IgGSD diagnosis was 7 y and that the duration of frequent/severe respiratory tract infection before IgGSD diagnosis was not significantly associated with routine clinical and laboratory variables, including referring physician specialties. We discuss our findings in the context of previous reports of IgGSD diagnosis in adults.

## Methods

### Ethics statement

This work was performed according to the principles of the Declaration of Helsinki [9]. Western Institutional Review Board provided an exemption under 45 CFR 46.101(b)(4) pertinent to this study on 18 October 2018 (submission 2535878–44170911; 2 October 2018). Obtaining informed consent was not required because this study involved retrospective chart review and analyses of observations recorded in routine medical care.

### Patient selection

We studied consecutive unrelated self-identified non-Hispanic white adults (ages ≥18 y) referred to a single outpatient referral practice because they had frequent or severe upper or lower respiratory tract infection and were diagnosed to have IgGSD [1–3] before 2 October 2018. Subnormal IgG4 level alone was not a criterion for IgGSD diagnosis [1–3]. We compiled referring physician specialties. Upper respiratory tract infection was defined as reports of sinusitis, otitis media, mastoiditis, pharyngitis, and tonsillitis. Lower respiratory tract infection was defined as reports of bronchitis, pneumonia, and bronchiectasis. Frequent infection was defined as four or more episodes per year that required antibiotic therapy. Severe infection was defined as any infection that required in-hospital treatment.

We excluded adults with Ig deficiency other than IgGSD [3], including: common variable immunodeficiency, ataxia-telangiectasia, X-linked agammaglobulinemia; hypogammaglobulinemia associated with acute infection, monoclonal gammopathy, B-cell neoplasms or other malignancies, organ transplantation, immunosuppressive therapy, anti-cancer chemotherapy, carbamazepine therapy, or increased Ig loss; infection with parasites, *Mycobacterium* sp., or human immunodeficiency virus; and incomplete evaluations.

### Duration of infection and age at onset of infection

We compiled reports of duration of frequent or severe respiratory tract infection before IgGSD diagnosis. Age at onset of frequent or severe infection was defined as the difference between age at IgGSD diagnosis and reported duration of frequent or severe respiratory tract infection before IgGSD diagnosis.

### Other conditions

We classified diabetes according to the criteria of the American Diabetes Association [10]. Autoimmune condition(s) and atopy (allergic asthma, allergic rhinitis, and allergic dermatitis/eczema) were diagnosed by referring physicians. Other allergy included urticaria, angioedema, or anaphylaxis [2]. We defined a dichotomous corticosteroid therapy variable as previously described [2]. Body mass index was computed as kg/m^2^.

### Laboratory methods

Serum Ig levels were measured using standard methods (Laboratory Corporation of America, Burlington, NC, USA) before IgG replacement therapy was initiated. We defined mean ± 2 standard deviations (SD) as reference ranges for all Ig measurements [2,11]. Reference ranges are: IgG 7.0–16.0 g/L (700–1600 mg/dL); IgG1 4.2–12.9 g/L (422–1292 mg/dL); IgG2 1.2–7.5 g/L (117–747 mg/dL); IgG3 0.4–1.3 g/L (41–129 mg/dL); IgG4 0–2.9 g/L (1–291 mg/dL); IgA 700–4000 mg/L (70–400 mg/dL); and IgM 400–2300 mg/L (40–230 mg/dL). Subnormal Ig levels were defined as those below the corresponding lower reference limits and were documented twice in all adults at times they did not have acute infection. We used the second IgG subclass values for the present analyses. Elevated Ig levels were defined as those above the corresponding lower reference limit.

Blood lymphocyte subsets were measured using flow cytometry (Laboratory Corporation of America, Burlington, NC, USA). Reference ranges (mean ± 2 SD) are: CD19+ 12–645 cells/μL; CD3+/CD4+ 359–1,519 cells/μL; CD3+/CD8+ 109–897 cells/μL; and CD56+/CD16+ 24–406 cells/μL. Subnormal levels were defined as those below the corresponding lower reference limit.

HLA-A and -B types and haplotypes were detected using low-resolution DNA-based typing (polymerase chain reaction/sequence-specific oligonucleotide probe) [12]. Control subjects were 751 unrelated white subjects from Alabama who underwent HLA-A and -B haplotyping to establish paternity [13]. For analyses, we selected the three most common haplotypes that occur with greater prevalence in Alabama non-Hispanic white adults with IgGSD than in control subjects (A*01,B*08; A*02,B*44; and A*03,B*07) [2,12].

### Statistics

The dataset for analyses consisted of complete observations on 300 adults. All data underlying the findings reported in this work are provided in a Supporting Information file (S1 File). All age at diagnosis, infection duration, and age at infection onset data were expressed as the nearest whole year. IgG4 levels <1 mg/dL were imputed as 0.5 mg/dL. Descriptive data are displayed as enumerations, percentages, mean ± 1 SD, or median (range). We evaluated continuous data for normality using d'Agostino's test and normal probability plots. We performed backward stepwise regressions on age at IgGSD diagnosis, duration of frequent or severe infection before IgGSD diagnosis, and age at onset of frequent or severe infection using these independent routine clinical and laboratory variables, as appropriate: sex; age at diagnosis; diabetes; autoimmune condition(s); atopy; allergy; corticosteroid use; body mass index; serum Ig isotype levels; blood lymphocyte subsets; HLA-A*01,B*08; -A*02,B*44; and -A*03,B*07 haplotype positivity (heterozygosity or homozygosity); and referring physician specialties. We defined values of p <0.05 to be significant. Analyses were performed with Excel 2000 (Microsoft Corp., Redmond, WA, USA) and GB-Stat (v. 10.0, 2003, Dynamic Microsystems, Inc., Silver Spring, MD, USA).

## Results

### General characteristics of 300 adults with IgGSD

Mean age at diagnosis was 50 ± 12 y (median 50 y (range 19–79)). There were 247 women (82.3%). Otolaryngologists, primary care physicians, rheumatologists, and pulmonologists referred 29.3%, 28.7%, 25.0%, and 9.3% of adults, respectively (92.3% in aggregate). Other referring physicians were endocrinologists, gastroenterologists, neurologists, infectious disease specialists, gynecologists, and cardiologists (in decreasing order).

Thirty-one adults (10.3%) had type 2 diabetes. One hundred and fifteen adults (38.3%) were diagnosed to have an autoimmune condition(s). Atopy, other allergy, and corticosteroid therapy occurred in 24.0%, 27.3%, and 18.7% of adults, respectively. Median body mass index was 27.9 kg/m^2^ (range 16.3–68.6).

Subnormal blood levels of CD19+, CD3+/CD4+, CD3+/CD8+, and CD56+/CD16+ cells were observed in 1.0%, 5.0%, 1.7%, and 3.3% of adults, respectively. Elevated blood levels of CD19+, CD3+/CD4+, CD3+/CD8+, and CD56+/CD16+ cells were observed in 2.3%, 1.2%, 3.7%, and 1.0% of adults, respectively.

IgG immunophenotypes are displayed in Table 1. Subnormal IgG1, IgG1/IgG3, and IgG3 accounted for 261 immunophenotypes (87.0%). Positivity for HLA haplotypes A*01,B*08; A*02,B*44; and A*03,B*07 was observed in 23.0%, 20.3%, and 11.0% of adults, respectively.

**Table 1:** Subnormal IgG subclass immunophenotypes in 300 adults with IgG subclass deficiency^a^^,^^b^.

Immunophenotype	Percent of 300 adults (n)
G1 alone	25.0 (75)
G2 alone	1.3 (4)
G3 alone	34.3 (103)
G1/G2	0.7 (2)
G1/G3	27.7 (83)
G1/G4	0.7 (2)
G2/G3	3.0 (9)
G3/G4	1.3 (4)
G1/G2/G3	4.3 (13)
G1/G2/G4	1.3 (4)
G1/G3/G4	0.3 (1)

^a^Subnormal IgG subclass levels were defined as those >2 standard deviations below the respective means: IgG1 <4.2 g/L (<422 mg/dL); IgG2 <1.2 g/L (<117 mg/dL); IgG3 <0.4 g/L (<41 mg/dL); and IgG4 0 g/L (<1 mg/dL).

^b^Total serum IgG was subnormal in 123 adults (41.0%) and elevated in 4 adults (1.3%). Twenty-three adults (7.7%) had subnormal serum IgA (reference 700–4000 mg/L (70–400 mg/dL)). Ten adults (3.3%) had elevated serum IgA levels. Forty-two adults (14.0%) had subnormal serum IgM (reference 400–2300 mg/L (40–230 mg/dL)). Twenty-one adults (7.0%) had elevated serum IgM levels.

### Age at IgGSD diagnosis

**Fig 1:**
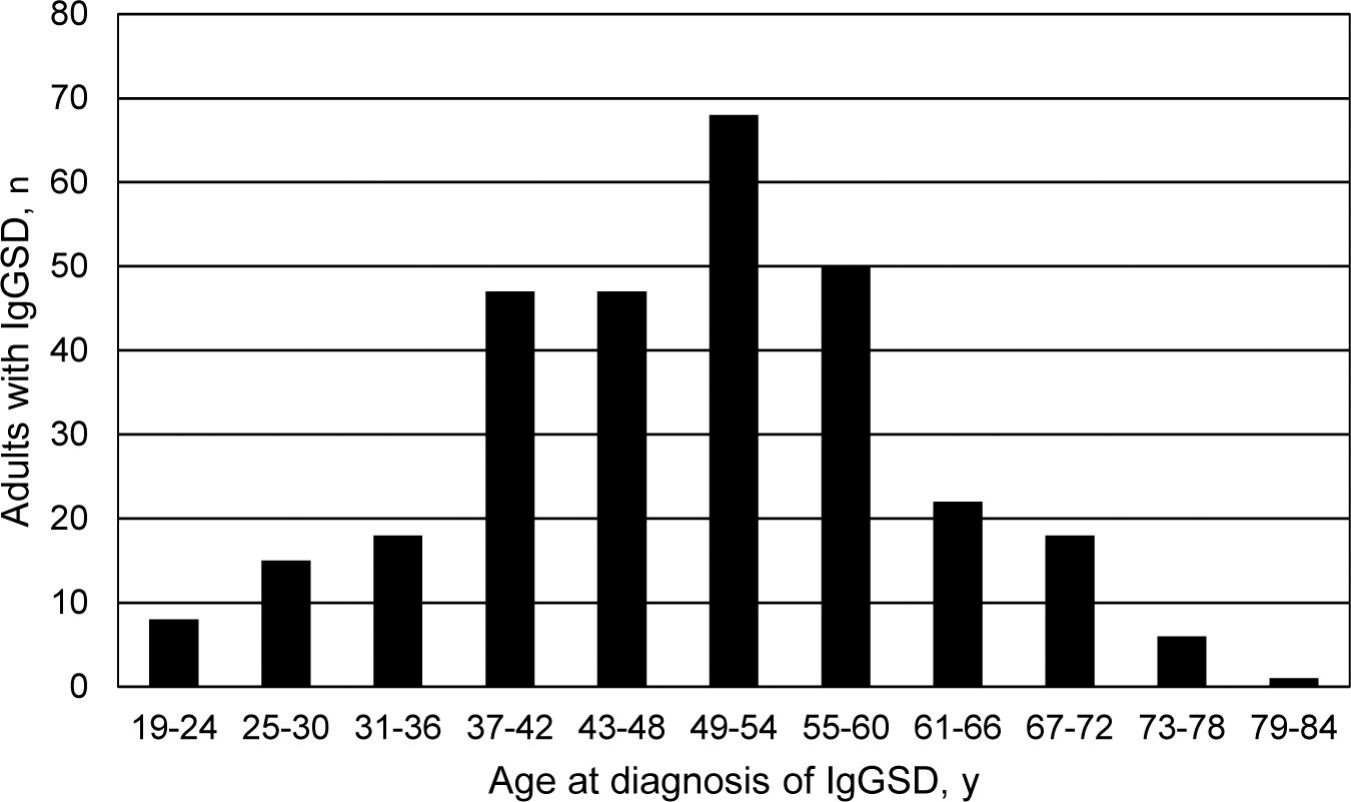
Frequency histogram of age at diagnosis of IgG subclass deficiency in 300 unrelated non-Hispanic white adults. Mean age of diagnosis was 50 ± 12 y (median 51 y (19–79)). These data are normally distributed.

These data were normally distributed (Fig 1). The age range 49–54 y included 68 adults (22.7%). The age range 43–60 y included 165 adults (55.0%). The age range 37–66 y included 234 adults (78.0%) (Fig 1). The age range 25–78 y included 291 adults (97.0%).

### Duration of infection before IgGSD diagnosis

**Fig 2:**
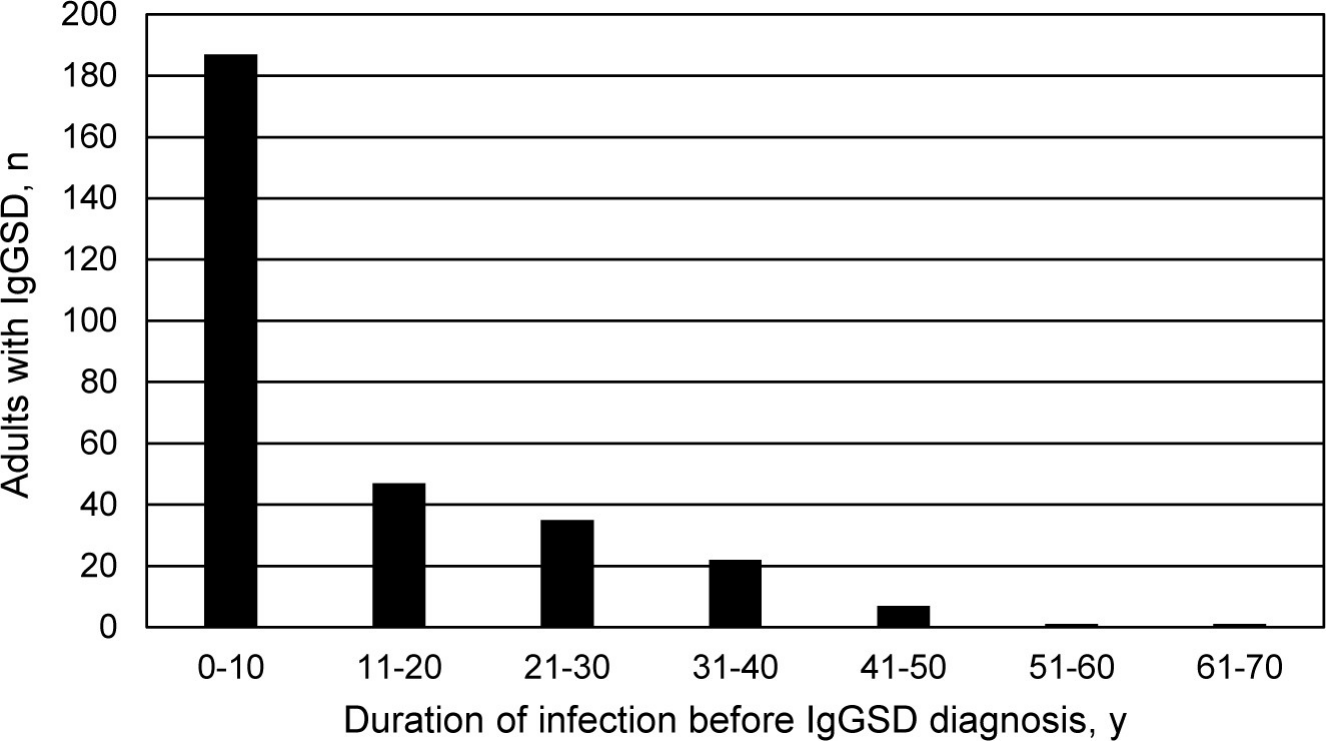
Frequency histogram of duration of frequent or severe respiratory tract infection in 300 unrelated non-Hispanic white adults before diagnosis of IgG subclass deficiency. Mean duration of infection before diagnosis of IgG subclass deficiency was 12 ± 13 y (median 7 y (range 1–66)). These data are not normally distributed.

These data were not normally distributed (Fig 2). Mean duration of frequent or severe respiratory tract infection before IgGSD diagnosis was 12 ± 13 y (median 7 y (range 1–66)). Proportions of adults who reported that they experienced frequent or severe respiratory tract infection ≤1 y and ≤2 y before IgGSD diagnosis were 15.0% and 28.0%, respectively. Sixty-four percent of adults experienced frequent or severe respiratory tract infection for ≤10 y before IgGSD diagnosis. Nine adults (3.0%) experienced frequent or severe respiratory tract infection for >40 y before IgGSD diagnosis.

### Age at onset of infection

**Fig 3:**
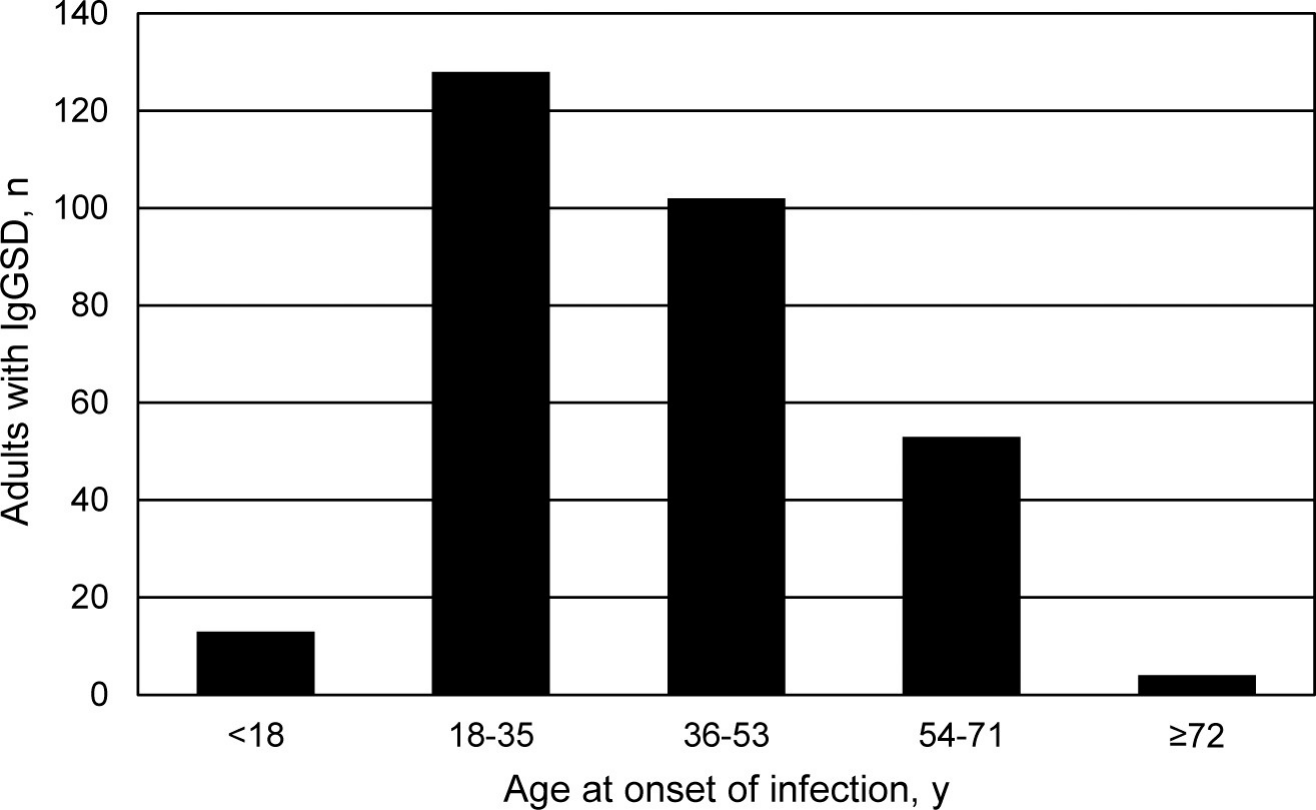
Frequency histogram of age at onset of frequent or severe respiratory tract infection in 300 unrelated non-Hispanic white adults subsequently diagnosed to have IgG subclass deficiency. Mean age at onset of infection was 38 ± 16 y (median 38 y (range 4, 76)). These data are not normally distributed.

Age at onset of frequent or severe respiratory tract infection data were not normally distributed (Fig 3). Mean age at onset of infection was 38 ± 16 y (median 38 y (range 4, 76)). One-hundred and forty-one adults (47.0%) reported that they experienced frequent or severe respiratory infection at ages ≤35 y. Thirteen adults (4.3%; 2 men, 11 women) reported that they experienced frequent or severe respiratory infection before age 18 y (range 4–17 y). The remaining 287 (95.7%) adults reported that they experienced frequent or severe respiratory infection at age ≥18 y.

### Regression on age at IgGSD diagnosis

We performed a backward stepwise variable regression on age at IgGSD diagnosis using these independent variables: sex; diabetes; autoimmune condition(s); atopy; allergy; corticosteroid use; body mass index; serum Ig isotype levels; blood lymphocyte subsets; three HLA-A and -B haplotypes; and referring physician specialties. This regression revealed no significant associations.

### Regression on duration of infection before IgGSD diagnosis

We performed a backward stepwise variable regression on duration of infection before IgGSD diagnosis using these independent variables: sex; age at diagnosis; diabetes; autoimmune condition(s); atopy; allergy; corticosteroid use; body mass index; serum Ig isotype levels; blood lymphocyte subsets; three HLA-A and -B haplotypes; and referring physician specialties. This regression revealed no significant associations.

### Regression on age at onset of infection

We performed a backward stepwise variable regression on age at onset of frequent or severe respiratory tract infection using these independent variables: sex; age at diagnosis; diabetes; autoimmune condition(s); atopy; allergy; corticosteroid use; body mass index; serum Ig isotype levels; blood lymphocyte subsets; three HLA-A and -B haplotypes; and referring physician specialties. This regression revealed a single positive association: age at diagnosis (p <0.0001). This regression accounted for 42.2% of the deviance of age at onset of infection (ANOVA p of regression <0.0001).

## Discussion

The mean age (range) at IgGSD diagnosis in the present adults (50 y (19–79)) is consistent with those of three other adult IgGSD cohorts from The Netherlands and US: 49 y (33–70) [14]; 51 y (18–89) [12]; and 47 y (17–71) [15], respectively. In 59 Korean adults with either asthma or chronic obstructive pulmonary disease, the mean age at subsequent IgGSD diagnosis was 61 ± 16 y [6]. Our age at IgGSD diagnosis data were normally distributed, consistent with a previous report that included 398 Alabama adults with IgGSD [2]. In other studies, age at IgGSD diagnosis in adults classified by IgG subclass immunophenotypes was not significantly associated with routine clinical and laboratory variables [16,17]. Because the present regression on age at IgGSD diagnosis also revealed no significant associations with the same clinical and laboratory variables, it is likely that heritable or acquired factors or health care delivery features other than those we studied influence age at IgGSD diagnosis in adults.

Duration of frequent or severe respiratory tract infection before IgGSD diagnosis in the present adults was great (mean 12 y, median 7 y). In a cohort of English adults, the median duration of frequent or severe respiratory tract infection before diagnosis of IgGSD or specific antibody deficiency was 11 y [5]. Most Korean adults with asthma or chronic obstructive pulmonary disease were diagnosed to have IgGSD more than 5 y after initial treatment of their respective pulmonary conditions [6]. These and other reports [4,7] substantiate that many adults with IgGSD experience long intervals of frequent or severe respiratory tract infection before IgGSD diagnosis.

Frequent or severe respiratory tract infection is the most common manifestation of undiagnosed IgGSD [4,18–20]. By definition, all of the present adults had numerous out-patient or in-hospital physician encounters for management of respiratory tract infection before referral and IgGSD diagnosis, but multivariable regression on duration of infection revealed no significant associations with routine clinical and laboratory variables. It is plausible that frequent or severe respiratory tract infection in some adults with undiagnosed IgGSD was interpreted as a sequel of diabetes [21,22], chronic sinusitis or rhinosinusitis [19,23], autoimmune condition(s) [24,25], asthma [26–28], allergic rhinitis [29], or chronic obstructive pulmonary disease [4,6,18]. Nonetheless, these observations also suggest that lack of knowledge about IgGSD among physicians who treat adults with respiratory tract infection may inadvertently increase the duration of frequent or severe respiratory tract infection before IgGSD diagnosis.

Diabetes was diagnosed in more than 10% of the present adults with IgGSD, a proportion similar to that of adults ages ≥18 y with diabetes in the general Alabama population (12%) [30]. Diabetes was not significantly associated with the age of onset or duration of frequent or severe respiratory tract infection in the present cohort, although this does not exclude a role of diabetes in antigen response, immunoglobulin synthesis, or infection susceptibility in adults with or without IgGSD. In a prospective study of 247 adults with diabetes, 48 adults had either a history of infections or a globulin level <2.2 g/dL [22]. The prevalence of subnormal IgG, IgA, or IgM, alone or in combination, was significantly higher in the adults with diabetes than in the general population and could explain in part the greater susceptibility of adults with diabetes to infection [22].

Physicians of different specialties treat patients with frequent or severe respiratory tract infection. Otolaryngologists, primary care physicians, rheumatologists, and pulmonologists referred 92.3% of the present adults, consistent with results of a previous study of adults with IgGSD or common variable immunodeficiency [2]. In the present study, duration of frequent or severe respiratory tract infection or age at onset of frequent or severe respiratory tract infection was not significantly associated with referring physician specialty. In another study, allergists and clinical immunologists, otorhinolaryngologists, and pulmonologists in aggregate referred 55% and primary care physicians referred 17% of 244 Canadian adults with immune deficiency diagnosed in a university center for adults with primary immune deficiency [31]. Pre-referral measurement of serum Ig levels was performed in only one-third of the 244 Canadian adults [31]. In a 1989 report from northwest England, the median delay in diagnosis of primary antibody deficiency in adults was 5.5 y [7]. After introduction of UK national diagnosis and treatment guidelines in 1995, median diagnostic delay in adults and children with primary antibody deficiency decreased to 2 y by 2005, although the proportion of referrals from primary care physicians remained low [8]. These observations suggest that educating physicians of many specialties about clinical and laboratory characteristics of immune deficiency could reduce delays in IgGSD diagnosis in adults.

Ninety-six percent of the present adults reported that they experienced frequent or severe respiratory tract infection at ages ≥18 y, suggesting that IgGSD in most adults becomes clinically manifest in adulthood. Almost half of the present adults reported that they first experienced frequent or severe respiratory tract infection at ages ≤35 y. Our multivariable regression on age at onset of infection before IgGSD diagnosis revealed a significant positive association with age at IgGSD diagnosis only. This suggests that older adults are diagnosed to have IgGSD after longer intervals of frequent or severe respiratory tract infection than younger adults, after adjustment for other variables.

The principal significance of this study is that many of the present adults with frequent or severe respiratory tract infection experienced a delay of many months or years before the underlying cause of their infections was diagnosed, yet most of them had repeated healthcare encounters for treatment of respiratory tract infection before they were evaluated with Ig measures [32]. Consequently, effective infection prophylaxis with IgG [33] and other management were delayed, thus increasing infection-associated morbidity and expense.

A strength of this study is that our sample size provides statistical power to detect significant independent associations with age at IgGSD diagnosis, duration of frequent or severe respiratory tract infection before IgGSD diagnosis, and age at onset of frequent or severe respiratory tract infection in multivariable models. Limitations of this study include lack of serum Ig levels measured in the present adults long before IgGSD diagnosis and possible inaccuracy of reports of duration of frequent or severe respiratory tract infection before IgGSD diagnosis. Another limitation is the lack of observations on a) ostensibly healthy, age- and sex-matched control subjects from the general non-Hispanic white population and b) other control subjects who had frequent or severe respiratory tract infection without evidence of IgGSD or other antibody deficiency. Evaluation of other heritable or acquired factors or health care delivery features that may influence duration of frequent or severe respiratory tract infection in adults before IgGSD diagnosis and morbidity associated with delayed diagnosis of IgGSD was beyond the scope of this study.

## Conclusions

Median duration of frequent or severe respiratory tract infection in adults before IgGSD diagnosis was 7 y. Older adults may be diagnosed to have IgGSD after longer intervals of infection than younger adults. Duration of frequent or severe respiratory tract infection before IgGSD diagnosis was not significantly associated with routine clinical and laboratory variables, including referring physician specialties.

## Supporting information

S1 File Observations on 300 adults with IgG subclass deficiency. (XLSX)

## References

[1] Conley ME, Notarangelo LD, Etzioni A. Diagnostic criteria for primary immunodeficiencies. Representing PAGID (Pan-American Group for Immunodeficiency) and ESID (European Society for Immunodeficiencies). Clin Immunol. 1999;93:190–197. 10.1006/clim.1999.4799 10600329

[2] Barton JC, Bertoli LF, Barton JC. Comparisons of CVID and IgGSD: referring physicians, autoimmune conditions, pneumovax reactivity, immunoglobulin levels, blood lymphocyte subsets, and HLA-A and -B typing in 432 adult index patients. J Immunol Res. 2014;2014:542706 10.1155/2014/542706 25295286 PMC4180398

[3] Bousfiha A, Jeddane L, Picard C, Ailal F, Bobby GH, Al-Herz W, et al The 2017 IUIS Phenotypic Classification for Primary Immunodeficiencies. J Clin Immunol. 2018;38:129–143. 10.1007/s10875-017-0465-8 29226301 PMC5742599

[4] Popa V: Airway obstruction in adults with recurrent respiratory infections and IgG deficiency. Chest. 1994;105:1066–1072. 8162726 10.1378/chest.105.4.1066

[5] Duraisingham SS, Buckland M, Dempster J, Lorenzo L, Grigoriadou S, Longhurst HJ. Primary vs. secondary antibody deficiency: clinical features and infection outcomes of immunoglobulin replacement. PLoS One. 2014;9:e100324 10.1371/journal.pone.0100324 24971644 PMC4074074

[6] Kim JH, Park S, Hwang YI, Jang SH, Jung KS, Sim YS, et al Immunoglobulin G subclass deficiencies in adult patients with chronic airway diseases. J Korean Med Sci. 2016;31:1560–1565. 10.3346/jkms.2016.31.10.1560 27550483 PMC4999397

[7] Blore J, Haeney MR. Primary antibody deficiency and diagnostic delay. BMJ. 1989;298:516–517. 10.1136/bmj.298.6672.516 2495090 PMC1835789

[8] Seymour B, Miles J, Haeney M. Primary antibody deficiency and diagnostic delay. J Clin Pathol. 2005;58:546–547. 10.1136/jcp.2004.016204 15858130 PMC1770645

[9] World Medical Association Declaration of Helsinki: ethical principles for medical research involving human subjects. JAMA. 2013;310:2191–2194. 10.1001/jama.2013.281053 24141714

[10] American Diabetes Association: Diagnosis and classification of diabetes mellitus. Diabetes Care. 2014;37 Suppl 1:S81–S90. 24357215 10.2337/dc14-S081

[11] Schauer U, Stemberg F, Rieger CH, Borte M, Schubert S, Riedel F, et al IgG subclass concentrations in certified reference material 470 and reference values for children and adults determined with The Binding Site reagents. Clin Chem. 2003;49:1924–1929. 14578325 10.1373/clinchem.2003.022350

[12] Barton JC, Bertoli LF, Acton RT. HLA-A and -B alleles and haplotypes in 240 index patients with common variable immunodeficiency and selective IgG subclass deficiency in central Alabama. BMC Med Genet. 2003;4:3 10.1186/1471-2350-4-3 12803653 PMC166147

[13] Barton JC, Acton RT. HLA-A and -B alleles and haplotypes in hemochromatosis probands with HFE C282Y homozygosity in central Alabama. BMC Med Genet. 2002;3:9 10.1186/1471-2350-3-9 12370085 PMC137582

[14] Van Kessel DA, Horikx PE, Van Houte AJ, De Graaff CS, Van Velzen-Blad H, Rijkers GT. Clinical and immunological evaluation of patients with mild IgG1 deficiency. Clin Exp Immunol. 1999;118:102–107. 10.1046/j.1365-2249.1999.01023.x 10540166 PMC1905395

[15] Abrahamian F, Agrawal S, Gupta S. Immunological and clinical profile of adult patients with selective immunoglobulin subclass deficiency: response to intravenous immunoglobulin therapy. Clin Exp Immunol. 2010;159:344–350. 10.1111/j.1365-2249.2009.04062.x 20015274 PMC2819500

[16] Barton JC, Bertoli LF, Barton JC, Acton RT. Selective subnormal IgG3 in 121 adult index patients with frequent or severe bacterial respiratory tract infections. Cell Immunol. 2016;299:50–57. 10.1016/j.cellimm.2015.09.004 26410396

[17] Barton JC, Bertoli LF, Barton JC, Acton RT. Selective subnormal IgG1 in 54 adult index patients with frequent or severe bacterial respiratory tract infections. J Immunol Res. 2016;2016:1405950 10.1155/2016/1405950 27123464 PMC4830719

[18] O'Keeffe S, Gzel A, Drury R, Cullina M, Greally J, Finnegan P. Immunoglobulin G subclasses and spirometry in patients with chronic obstructive pulmonary disease. Eur Resp J. 1991;4:932–936. 1783083

[19] Schwitzguébel AJ, Jandus P, Lacroix JS, Seebach JD, Harr T. Immunoglobulin deficiency in patients with chronic rhinosinusitis: Systematic review of the literature and meta-analysis. J Allergy Clin Immunol. 2015;136:1523–1531. 10.1016/j.jaci.2015.07.016 26329513

[20] Berger M, Geng B, Cameron DW, Murphy LM, Schulman ES. Primary immune deficiency diseases as unrecognized causes of chronic respiratory disease. Respir Med. 2017;132:181–188. 10.1016/j.rmed.2017.10.016 29229095

[21] Peleg AY, Weerarathna T, McCarthy JS, Davis TM. Common infections in diabetes: pathogenesis, management and relationship to glycaemic control. Diabetes Metab Res Rev. 2007;23:3–13. 10.1002/dmrr.682 16960917

[22] Mazer NA, Welbourn D, Bernstein RK, Rubinstein A. Immunoglobulin deficiency in patients with diabetes mellitus. J Allergy Clin Immunol. 2011;127:AB12.

[23] Odat H, Alqudah M. Prevalence and pattern of humoral immunodeficiency in chronic refractory sinusitis. Eur Arch Otorhinolaryngol. 2016;273:3189–3193. 10.1007/s00405-016-3981-x 26975445

[24] Arkwright PD, Abinun M, Cant AJ. Autoimmunity in human primary immunodeficiency diseases. Blood. 2002;99:2694–2702. 11929755 10.1182/blood.v99.8.2694

[25] Ercolini AM, Miller SD. The role of infections in autoimmune disease. Clin Exp Immunol. 2009;155:1–15. 10.1111/j.1365-2249.2008.03834.x 19076824 PMC2665673

[26] Martin RJ, Kraft M, Chu HW, Berns EA, Cassell GH. A link between chronic asthma and chronic infection. J Allergy Clin Immunol. 2001;107:595–601. 10.1067/mai.2001.113563 11295645

[27] Iikura M, Hojo M, Koketsu R, Watanabe S, Sato A, Chino H, et al The importance of bacterial and viral infections associated with adult asthma exacerbations in clinical practice. PLoS One. 2015;10:e0123584 10.1371/journal.pone.0123584 25901797 PMC4406689

[28] Mao W, Cui EH. Distribution of pathogens causing nosocomial infection in patients with bronchial asthma. Genet Mol Res. 2015;14:16146–16150. 10.4238/2015.December.8.3 26662406

[29] Alho OP, Karttunen R, Karttunen TJ. Nasal mucosa in natural colds: effects of allergic rhinitis and susceptibility to recurrent sinusitis. Clin Exp Immunol. 2004;137:366–372. 10.1111/j.1365-2249.2004.02530.x 15270854 PMC1809099

[30] Centers for Disease Control and Prevention. Diabetes 2017 Report Card. Centers for Disease Control and Prevention, US Dept of Health and Human Services. 2018 Atlanta, GA, Centers for Disease Control and Prevention, US Dept of Health and Human Services.

[31] Srinivasa BT, Alizadehfar R, Desrosiers M, Shuster J, Pai NP, Tsoukas CM. Adult primary immune deficiency: what are we missing? Am J Med. 2012;125:779–786. 10.1016/j.amjmed.2012.02.015 22682795

[32] Parker AR, Skold M, Ramsden DB, Ocejo-Vinyals JG, Lopez-Hoyos M, Harding S. The clinical utility of measuring IgG subclass immunoglobulins during immunological investigation for suspected primary antibody deficiencies. Lab Med. 2017;48:314–325. 10.1093/labmed/lmx058 29126302 PMC5907904

[33] Albin S, Cunningham-Rundles C. An update on the use of immunoglobulin for the treatment of immunodeficiency disorders. Immunotherapy. 2014;6:1113–1126. 10.2217/imt.14.67 25428649 PMC4324501

